# Effects of Mindfulness-Based Elder Care (MBEC) on symptoms of depression and anxiety and spiritual well-being of institutionalized seniors with disabilities: a randomized controlled trial

**DOI:** 10.1186/s12877-023-04220-6

**Published:** 2023-08-18

**Authors:** Yvonne Hsiung, Yi-Heng Chen, Li-Chan Lin, Yu-Han Wang

**Affiliations:** 1 Department of Nursing, Mackay Medical College, New Taipei City, Taiwan; 2 Department of Nursing and Institute of Geriatric Welfare Technology and Science, Mackay Medical College, 46, Sec. 3, Zhongzheng Rd., Sanzhi Dist., New Taipei City, Taiwan; 3 Department of Nursing, Asia University, Taichung, Taiwan; 4 Senior Welfare Group of Social and Family Affairs Administration, Ministry of Health and Welfare, Taipei City, Taiwan

**Keywords:** Disabilities, Residential care facility, Mental health, Spiritual well-being, Mindfulness mediation, Seniors

## Abstract

**Background:**

Despite the need to incorporate seniors from various settings into mindfulness-based empirical research, issues of geriatric frailties and non-compliance remain. This study aimed to evaluate the effects of a mindfulness-based elder care (MBEC) program on mental health and spiritual well-being among seniors with disabilities in long-term care residential settings.

**Methods:**

This single-blind, randomized controlled trial (RCT) randomly assigned seventy-seven participants into an MBEC group or control group of an eight-week MBEC program. Participants were assessed every four weeks at baseline (T_0_), mid-intervention (T_1_), post-intervention (T_2_) and follow-up (T_3_) using the Geriatric Depression Scale Short Form (GDS-SF), the State-Trait Anxiety Inventory (STAI) and the Spiritual Well-Being Scale (SWBS), respectively.

**Results:**

Linear mixed model (LMM) showed that MBEC participants’ mental health improved significantly after completing the intervention; compared with controls, the MBEC group exhibited significantly lower anxiety (state-anxiety at T_2_; trait-anxiety at T_2_ and T_3_) and fewer depressive symptoms. Spiritual well-being was also significantly enhanced compared to that in the control group.

**Conclusions:**

MBEC has positive effects on both mental health and spiritual well-being outcomes among seniors with disabilities. In long-term care facilities, seniors with abilities have the potential to adhere to and engage in activities of a mindfulness-based intervention. This low risk, easily accessible, and effective 8-week program is recommended to be integrated into regular long-term care institutional routines.

**Trial registration:**

This study was registered with Clinical Trial Registry (ClinicalTrials.gov – U.S. National Library of Medicine #NCT05123261. Retrospectively registered on 07/04/2021.). The CONSORT 2010 guidelines were used in this study for properly reporting how the randomized trial was conducted.

## Introduction

Population aging is currently a worldwide phenomenon. Senior members of society are prone to chronic diseases, adding extra complications to pre-existing disabilities. Permanent relocation to long-term care (LTC) facilities becomes increasingly common among dependent seniors, due to the inability of family caregivers to provide complex 24-h care. While seniors with disabilities are bound to stay in a restrictive environment, the stressful event of institutionalization typically results in loss of independence, privacy, and shared social interaction with loved ones [1] and may further impact their mental health and spiritual well-being. However, this impact of relocation at the later-stage in life may not be completely negative [2]. A recent qualitative study found that elderly Taiwanese, despite initial adaptation challenges, valued the tailored care in long-term care facilities and gradually adapted [3].

To address the multiple care needs of seniors with disabilities, holistic care models and interventions that address body, mind and spirit are specifically emphasized in residential care settings. The efficacy of mind-and-body approaches such as mindfulness-based interventions are well-supported across various disease and age populations to promote individual physical, psychological, and spiritual well-being [4, 5]. Kabat-Zinn [6] described that mindfulness arises through an individual’s deliberate focus on the present moment, including his/her sensations, bodily states, thoughts, and consciousness. Mindfulness-based interventions (MBIs) therefore have been established to facilitate formal (e.g., breathing, sitting, walking, body scan) or informal (e.g., mindfulness in everyday life) practices to cultivate moment-by-moment awareness of thoughts, feelings, bodily sensations [7]. The two most widely adopted MBIs, mindfulness-based stress reduction (MBSR) [8] and mindfulness-based cognitive therapy (MBCT) [9] have been shown to be efficacious, particularly in improving mental health, general health, and quality of life [10].

Practicing mindfulness has yielded noticeable physio-psycho-spiritual effectiveness through an individual’s bio-physiological pathway, adaptive psychological processes, and personal spiritual growth [7, 11–13]. A schematic model [14] showed the effects of the biological mechanism of meditation on cognition through body, brain, and emotions. Practicing mindfulness meditation, as an adaptive psychological process, also helped individuals to attain self-regulation of their affect, thus reducing stress [7]. Results from multiple systematic reviews and meta-analyses of randomized controlled trials and quasi-experiment studies [11–13] demonstrated positive impact of MBIs to enhance psychological well-being in various populations. Furthermore, recent evidence has suggested that mindfulness is uniquely effective in promoting meaningfulness [15] to increase spiritual growth. In this longitudinal waitlist-controlled study, the positive effects of Mindfulness-Based Stress Reduction (MBSR) on spirituality and posttraumatic growth (PTG) were strongly supported; among cancer participants (N = 135), their MBSR practice has facilitated a sense of meaning, peacefulness, connectedness, and personal growth that promoted their spirituality, PTG, and mindfulness, relative to controls [15]. Additionally, paying attention in the present moment, with an attitude of acceptance and “letting go,” seems to be associated with enhanced spirituality [16, 17]. A waitlist-controlled intervention study (*N* = 211) examined the timing of changes during mindfulness-based cancer recovery, and the result of sequential mediated effects informed our understanding. The state of mindfulness and emotional regulation could be mechanisms of MBIs to improve spirituality [16]. In addition, in a stress-reduction cross-sectional study, participants’ (*N* = 44) increased mindfulness attention and awareness trait were related to enhanced mindfulness state spirituality, less psychological distress, and fewer reported medical symptoms [17].

Mindfulness-based stress reduction (MBSR) programs [8] were initially introduced in 1994 [6] to help individuals focus on adopting the regular practice of meditative skills into everyday life. In the original MBSR program, participants were required to practice different techniques of mindfulness meditation, including body scan, sitting meditation, mindful eating and walking, and yoga exercise [18], with specific attempts to enhance participants’ self-awareness of their moment-to-moment emotional and physical state and to reduce their negative affect and stress. Several systematic reviews have reported the effectiveness of MBSR in improving geriatric physical and cognitive functioning, symptoms of stress, anxiety and depression, quality of life [19–21], and spiritual growth [15, 17].

Despite the need to incorporate seniors from various settings into future mindfulness-based empirical research, current MBSR curriculums may not be suitable for seniors in residential care settings because of their compromised cognition and insufficient concentration originating from physical, cognitive, and communication infirmities. McBee and colleagues responded to these concerns and developed the first mindfulness-based elder care (MBEC) program in the late 90 s [22]. Compared to the MBSR curriculum, activities designed in MBEC were modified and adapted for frail seniors with various comorbidities to include fewer participants, shorter sessions, simplifier practice, and less homework [23]. The MBEC group usually comprises 6 to 10 seniors and each weekly session lasts 45–60 min. Kabat-Zinn’s core mindfulness attitudes, including non-judging, patience, non-striving, acceptance, and letting go, were applied in a series of simple and objective forms of mindfulness meditation that could be performed conveniently on a bed or wheelchairs. The program components included sitting and walking meditation, mindful eating, diaphragmatic breathing, simplified body scan, guided imagery, (un)pleasant experiences, gentle yoga or stretching and lovingkindness meditation [22, 24].

Although MBEC, centering on the self-awareness of “here and now,” was readily acceptable among seniors and their caregivers, only a few studies have supported its effects on senior residents’ pain intensity and mental health outcomes (effect size = 0.45) [25]. Previous MBEC studies, often employing a single-group design, chose limited outcome variables and lacked longitudinal data [22, 24, 25]. Despite relevant literature is scarce, MBEC has shown acceptability among frail elderly and their senior caregivers [23, 26, 27]. A cost-effective, non-intrusive, and non-pharmacological MBEC study with rigorous design is therefore still required to evaluate effectiveness. In addition, to the best of our knowledge, no MBEC-related studies have been conducted among Chinese/Taiwanese seniors in residential care settings, and the effects of the MBEC on the spiritual component have not been thoroughly examined. The primary purpose of this study was to evaluate the effects of the MBEC intervention on the mental health and spiritual well-being of long-term care residents.

## Methods

### Study design

This 8-week randomized, single blind, controlled trial (RCT) (ClinicalTrials.gov ID: NCT05123261) was conducted in northern and central Taiwan after receiving approval of the study protocol granted by our hospital’s Internal Review Board (13MMHIS244). Effects of the MBEC intervention were evaluated with a pretest–posttest design at four time-points, including baseline, mid-intervention, post-intervention, and 4-week follow-up (T_0_, T_1_, T_2_, and T_3_).

### Participant recruitment

Senior residents of long-term care aged 65 years or older who met certain disability criteria were recruited. Inclusion criteria were: 1) lived in residential LTC institutions for at least three months; 2) had an activities of daily living (ADL) score under 100 according to the Barthel Index [28]; and 3) were able to communicate in Mandarin or Taiwanese. Excluded were senior residents with a history of major depression, severe sensory and/or cognitive impairment, and were unable to follow instructions during the program, judged by the institutional physicians and nurses.

To determine an adequate sample size, a priori two-sided power analyses were calculated to identify a statistically significant effect (α = 0.05) and power of 0.80. Based on the given effect size estimated by a prior study, the effect size of Cohen's d was set at 0.36 in G-Power 3.1. The least sample size estimate was 22 participants per group; and if allowing a 20% attrition rate, the final sample should include 52 eligible participants in total.

**Fig. 1:**
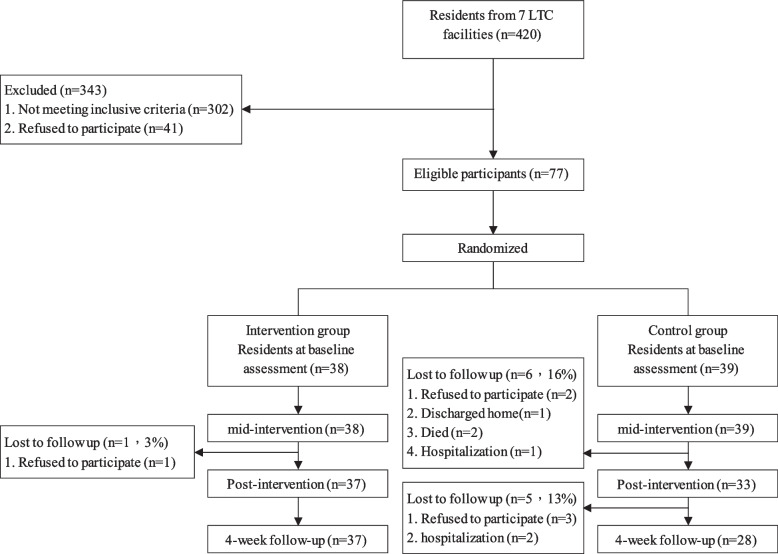
Flow diagram of the study

The flow of participants through each stage of study is shown in Fig. 1. Among a total of 420 residents from the seven LTC institutions who granted permission for this study to be conducted, 343 residents were excluded. Finally, seventy-seven (N = 77) were randomly assigned by the primary investigator using a random-number generator to the intervention group (IG) (n = 38) or the control group (CG) (n = 39). At 4 weeks after the intervention, 12 participants dropped out of the study due to personal illness, death, rejection, and/or hospitalization, resulting in a total 15% attrition rate for this study.

### Interventions

Before implementing the MBEC intervention, an 8-week pilot was conducted for six senior residents who met the equivalent criteria in a chosen LTC institution in northern Taiwan. Modifications were made according to practical advice from nine LTC and mindfulness practice experts regarding the sequence of weekly activities, the order of the modules, and the group format of end-session discussion.

The MBEC intervention consisted of eight weekly group sessions, containing 50-min MBEC lectures and activities each week (Table 1). Every MBEC session began with approximately 10 min of activity covering essential contents of mindfulness principles and reflections from previous sessions. IG participants were instructed to perform 30 min of mindfulness exercises, followed by a 10- to 20-min discussion before closing the session. The CG received usual care alone, provided by the LTC institutions; however, each CG participant had a weekly visit from the research team. Research assistants conducted a 10- to 15-min greeting and conversations unrelated to the mindfulness intervention. This technique was to ensure both the adherence and single-blinding of participants in the control group.

**Table 1:** Weekly topics and session activities of the MBEC intervention

Week	Topics	Group objectives	Session Contents/activities
1.	Increase awareness and live in the present moment	• Understand the goals and schedule of the intervention • Understand the meaning of living in the present moment and its impact on daily life	• Introduction to the MBEC program (10 min) • Mindful seeing and mindful eating (raisin exercise) (30 min) • Discussion (10 min)
2.	Organize thoughts:	• No thoughts about the past or the future • Be completely present in each moment	• Reflection of last week (10 min) • Diaphragmatic breathing (15 min) • Mediation (15 min) • Discussion (10 min)
3.	Be aware of your own body	• Understand your own body and feelings through stretching exercises	• Reflection of last week (10 min) • Gentle yoga (30 min) • Discussion (10 min)
4.	Seek for true self	• Accept and detect your own bodily conditions	• Reflection of last week (10 min) • Body scan (30 min) • Discussion (10 min)
5.	Integration of body and mind	• Connect your experience through feelings not thoughts	• Reflection of last week (10 min) • Walking and sitting meditation (30 min) • Discussion (10 min)
6.	Knowing what you like and dislike	• Be interested in unpleasant experiences, rather than avoid them	• Reflection of last week (10 min) • Sharing of (un)pleasant events and non-avoidance exercise (30 min) • Review of previous exercises/Discussion (10 min)
7.	Reality and thoughts	• Know that thoughts are just a psychological activity, not reality	• Reflection of last week (10 min) • Guided imagination (30 min) • Review of previous exercises/discussions (10 min)
8.	Take care of self and keep learning	• Be benevolent to self, no harshness • Allow things to be as they are • Do not attempt to change	• Reflection of seven weeks (10 min) • Loving-kindness mediation (15 min) • Review of previous exercises/discussions (15 min) • General feedback and sharing of the program

### Outcome measures

To evaluate the effectiveness of the MBEC intervention among seniors with disabilities living in residential LTC settings, the primary outcomes were participants’ depression and anxiety, and the secondary outcome was spiritual well-being.

### Instruments

Participants’ demographic information, length of stay in the institution, and religious beliefs were collected from the two groups at baseline. The Geriatric Depression Scale Short Form (GDS-SF), the State-Trait Anxiety Inventory (STAI) and the Spiritual Well-Being Scale (SWBS) were used to assess senior residents’ levels of depression, anxiety, and spiritual well-being, respectively.

#### Geriatric Depression Scale Short Form (GDS-SF)

The GDS-SF [29] was chosen for assessing depression among the senior participants. It is a brief, 15-item questionnaire in which participants are asked to respond by answering yes or no in reference to how they felt over the past week (5 items were negative). Scores of 0–4, 5–8, 9–11, and 12–15 are generally considered as normal, mild depression, moderate depression, and severe depression, respectively. The validity and reliability of this tool have been supported through both clinical practice and research, with sound validity to differentiate depressed from non-depressed adults [30]. In the Chinese version of the GDS-SF, Cronbach's α coefficient was 0.78, and convergent validity between the GDS Scale short form and long form was supported (r = 0.95) [31].

#### State-Trait Anxiety Inventory (STAI)

The STAI (Y form) is a commonly used measure of state and trait anxiety and distinguishes it from depressive syndromes [32]. It contains two separate subscales to measure state anxiety (20 items) and trait anxiety (20 items). The S-Anxiety subscale assesses the respondent’s feeling in that moment and the T-Anxiety subscale assesses how the respondent feels generally. All items in the two scales are rated on a 4-point scale (e.g., from “not at all” to “very much so”). The total score of each subscale ranged from 20–39, 40–59 and 60–80, indicating a low, moderate and high level of state or trait anxiety, respectively. Internal consistency coefficients for the scale ranged from 0.83 to 0.93, with test–retest reliability coefficients reported from 0.65 to 0.75 [32]. The psychometric properties of the STAI Chinese version (STAI-C) were measured in 306 Taiwanese outpatients with anxiety disorders. Cronbach's α coefficients for state and trait anxiety subscales were 0.91 and 0.92, and the criterion validity between the two subscales and the Hamilton Anxiety Rating Scale (HARS) [33] were supported (r = 0.69 for state and 0.74 for trait anxiety) [34].

#### Spiritual Well-Being Scale (SWBS)

The SWBS is a 20- item scale that measures an individual’s well-being and overall life satisfaction [35]. On two dimensions, items related to religious wellbeing measures were recognized as the well-being of his or her spiritual life in relation to their belief in God. Items related to existential well-being are about perceptions of life’s meaning and satisfaction in how well he or she is adjusted to self, community, and surroundings. All 20 items are rated on a 6-point scale (e.g., from “1-Always Disagreed” to “6-Always Agreed”). The total scores of 20–40, 41–99 and 100–120 reflect a sense of low, moderate and high spiritual well-being, respectively. Test–retest reliability coefficients for the total scale are reported from 0.82 to 0.99 and the concurrent validity of SWBS was established by significant correlations with other standard indicators of well-being [36]. The psychometric properties of the SWBS Mandarin version were measured in Taiwan, with a reported internal consistency of 0.91 and convergent validity of 0.73, consistent with the Spirituality Index of Well-Being Scales [37].

### Ethical considerations

Before approaching senior participants with disabilities, permissions were obtained from the participating residential institutions. To protect data integrity and respect participant privacy, all measurements were collected after obtaining signed consent for participation. Participants were also informed that no direct benefits were derived from voluntarily participating in this study, other than the three dollars (USD) of appreciation, as well as no known serious risks related to participation. Confidentiality was assured be ensured through the use of anonymizing techniques. Instant feedbacks after every session and preliminary results of this study were reported in anonymity at the group level to administration teams of participating institutions.

### Procedure

Every session of the MBEC program was led only by the primary investigator and research assistants (RAs), who were community nurses specializing in LTC practice and intensively trained by the Taiwanese Mindfulness Association, receiving MBEC certification. Held in a 10-person consultation room, one trained research assistant led participants’ group activities during the entire program. Additionally, senior residents participating in the IG were closely monitored by the same RA, and after each weekly session, telephone interviews were conducted twice a week to evaluate participants’ practice of diaphragmatic breathing in their institutions. Complementary booklets with mindfulness information, written reminders from session highlights, and encouraging messages were prepared by the RA, which could be shared with their families and friends, if needed. Moreover, to ensure participant compliance with the mindfulness practices, regular evaluations were conducted during the bi-weekly telephone interviews. The participants' mindfulness practice progress and feedback were monitored closely during each session, and continual encouragement was provided to promote adherence to the practice. This close monitoring served the purpose to confirm participants engaging in the mindfulness practices as guided.

All participant data were extracted from the institutional chart and face-to-face interviews with residents. Two RAs, each with a degree of Bachelor of Science in nursing, were trained to undertake data collection. The training courses included three hours of instruction in questionnaires introduction, conducting of interviews, and rating the responses. To ensure inter-rater agreement, two RAs took turns as interviewer or observer to complete 6 participants’ questionnaires from the pilot study, under the supervision of the researcher. The proportions of exact agreement of results obtained from all scales varied between 95%-100% for researcher-RA pairs. At the baseline (pretest T_0_), participants’ demographic and clinical characteristics were collected using clinical chart review. For outcome variables, geriatric depression, anxiety, and spiritual well-being of each participant were evaluated at four time-points using the structured questionnaires to conduct interviews independently in a private room.

### Statistical analysis

All data analysis was performed using the IBM Statistical Package for the Social Sciences (SPSS) 22.0 (SPSS Inc., Chicago, IL, USA). Senior residents’ characteristics and outcome variables were first depicted by descriptive statistics. Categorical Chi-square tests and independent t-tests were used to compare differences in characteristics between the two groups. To evaluate the effects of the intervention, a Linear mixed model (LMM) was employed. This model accounts for both within-group changes over time (main effects) and differences in these changes between the MBEC group and control group across time (interaction effects), exhibiting robust while handling missing data. The LMM primarily examined the interaction effects between the MBEC and control groups, along with the trajectory of change in outcomes of mental health and spiritual well-being over the course of the intervention and 4-week follow-up. The interaction between group (MBEC versus control) and time (baseline, mid-intervention, post-intervention, and 4-week follow-up) offered insights into the differential impact of the MBEC program compared to the control condition over time. Additionally, effect sizes were calculated for the outcome measures as well as the statistical significance. A p-value under 0.05 was considered statistically significant.

## Results

### Participants’ characteristics

The baseline characteristics of senior residents with disabilities are shown in Table 2. Females were dominant (60.5%), and the mean age of study participants was 78.95 (standard deviation [SD] = 8.10). The mean length of institutional stay was approximately 2.80 and 2.97 years for the IG and the CG, respectively. Participants’ mean Barthel Index score was 61.30 (SD = 32.04), indicating moderate to severe dependency. No significant differences were found between the two groups regarding their demographic characteristics.

**Table 2:** Baseline characteristics of long-term care institutional residents with disabilities

Characteristic	Intervention Group (N = 38)		Control Group (N = 39)		*X*^*2*^*/t*	*P*
	N (%)	M ± SD	N (%)	M ± SD		
Age		78.68 ± 7.97		79.21 ± 8.33	0.28	0.78
Gender					0.19	0.89
Male	15(39.5)		16(41.0)			
Female	23(60.5)		23(59.0)			
Marital Status					2.07	0.72
Married	10(26.3)		13(33.3)			
Single	4(10.5)		2(5.1)			
Widower	21(55.3)		20(51.3)			
Separated	0(0.0)		1(2.6)			
Divorced	3(7.9)		3(7.7)			
Religious affiliation					0.87	0.83
Buddhist/Taoist	23(60.5)		23(59.0)			
Protestant	1(2.6)		1(2.6)			
Catholics	8(21.1)		6(15.4)			
Atheist	6(15.8)		9(23.0)			
Education					5.13	0.40
Illiterate	8(21.1)		9(23.0)			
Literate/Uneducated	1(2.6)		4(10.3)			
Primary school	16(42.1)		11(28.2)			
Secondary school	6(15.8)		3(7.7)			
High school	5(13.1)		8(20.5)			
College/University	2(5.3)		4(10.3)			
Length of stay in institution (months)		33.55 ± 36.22		35.69 ± 31.20	0.28	0.78
Barthel Index		61.58 ± 31.88		61.03 ± 32.61	-0.08	0.94

*M* Mean, *SD* Standard deviation

### Mean scores of outcome variables from baseline to 4-week follow-up for IG and CG

Mean scores of outcome variables and the within-group effect of the MBEC intervention at four time-points are shown in Table 3. No significant differences between the IG and CG were found in the baseline outcome variables. IG participants’ mental health outcomes, including depression and state- and trait-anxiety were significantly improved between the pre-test and three post-testing time points (T_1,_ T_2_ and T_3_). Participants’ overall and existential spiritual well-being also improved significantly after completing the program (T_2_ and T_3_) (*p* < 0.001), along with a significant enhancement of the religious aspect of spiritual well-being at post-intervention (T_2_ vs. T_0_) (*p* < 0.05). On the other hand, CG participants also showed significant improvement in their mental health: their depression at the 4-week follow-up and state- and trait-anxiety were improved at 2 post-intervention time points compared to baseline (T_2_ vs. T_0_ & T_3_ vs. T_0_). However, throughout the study period, changes in CG participants’ spiritual well-being were insignificant at nearly all post-tests, except their religious aspect of spiritual well-being was significantly worse at post-intervention compared to baseline (T_2_ vs T_0_) (*p* < 0.001), resulting in a significant decrease of overall spiritual well-being (*p* < 0.01).

**Table 3:** Mean Scores of outcome variables from baseline to 4-week post-intervention follow-up

	Baseline(T0)			Mid-intervention(T1)			Post-intervention(T2)			4-week follow-up(T3)			β_0_	β_1_	β_2_	β_3_
	M ± SD			M ± SD			M ± SD			M ± SD				T_1 VS._ T_0_	T_2 VS._ T_0_	T_3 VS._ T_0_
**Intervention Group**																
Geriatric Depression	5.24 ± 3.41			4.32 ± 3.95			3.05 ± 3.46			3.61 ± 3.77			5.24^***^	-0.92^*^	-2.13^***^	-1.65^***^
State-anxiety	37.34 ± 12.02			33.34 ± 12.74			28.30 ± 15.53			29.00 ± 9.99			37.34^***^	-4.00^*^	-9.09^***^	-8.57^***^
Trait-anxiety	39.63 ± 12.69			35.50 ± 13.45			29.92 ± 10.42			28.58 ± 10.09			39.63^***^	-4.13^**^	-9.74^***^	-11.33^***^
Spiritual Well-being	82.29 ± 22.60			86.47 ± 22.75			92.22 ± 23.49			92.60 ± 23.76			82.29^***^	4.18	10.50^***^	9.63^***^
Religious well-being	44.76 ± 16.69			47.24 ± 16.70			47.95 ± 17.93			48.28 ± 17.19			44.76^***^	2.47	3.53^*^	2.91
Existential well-being	37.53 ± 12.39			39.24 ± 13.33			44.27 ± 11.50			43.83 ± 11.82			37.53^***^	1.71	6.54^***^	6.47^***^
**Control Group**																
Geriatric Depression	5.23 ± 3.98			4.76 ± 4.23			4.30 ± 4.50			4.07 ± 3.85			5.23^***^	-0.47	-0.97	-1.32^*^
State-anxiety	37.59 ± 14.06			34.33 ± 12.70			34.36 ± 12.33			32.82 ± 10.88			37.59^***^	-3.20	-3.30^*^	-5.15^**^
Trait-anxiety	35.87 ± 11.79			33.74 ± 11.22			32.70 ± 10.28			30.43 ± 9.20			35.87^***^	-2.12	-3.55^*^	-5.89^***^
Spiritual Well-being	75.38 ± 27.55			74.66 ± 27.32			65.42 ± 30.69			68.89 ± 30.45			75.38^***^	-1.03	-9.16^**^	-2.89
Religious well-being	36.49 ± 19.05			35.37 ± 18.23			27.12 ± 21.42			30.31 ± 22.23			36.48^***^	-1.48	-8.90^***^	-4.27
Existential well-being	38.90 ± 4.67			39.29 ± 13.63			38.30 ± 14.14			39.64 ± 13.47			38.89^***^	0.45	-0.18	1.68

*M* Mean, *SD* Standard deviation; Regression coefficient β0 is the baseline means for both group; regression coefficients β1, β2 and β3 indicates the time effect observed for mid-intervention (β1), post-intervention (β2) and 4-week follow-up (β3)

^*^p < 0.05; ^**^p < 0.01; ^***^p < 0.001

### Effects of intervention on outcome variables between groups

Results of the GLM models for mental health and spiritual well-being outcome variables are presented in Table 4. When examining intervention effects between the two groups on mental health outcomes of depression and anxiety, mixed-model analysis revealed that throughout the entire intervention, a positive trend of improvement was observed but no significant differences were found in improvement of depressive symptoms between the MBEC participants and controls. However, once the intervention was completed, the IG group exhibited significantly greater reduction in state-anxiety at T_2_ (effect size = 0.27) and trait-anxiety at both T_2_ (effect size = 0.14) and T_3_ (effect size = 0.11) compared to the control group. In terms of the effectiveness of the intervention to improve spiritual well-being, significant improvements were noted in the MBEC participants compared to the control group. Specifically, not only did the total SWBS scores significantly improve at T_2_ (effect size = 1.05) and T_3_ (effect size = 0.40), but also the two subscale scores, religious and existential well-being, showed significant increases. The religious well-being effect size was 0.47 at T_2_ and 0.42 at T_3_, and the existential well-being effect size was 0.23 at both T_2_ and T_3_ upon completion of the intervention.

**Table 4:** Linear mixed model of differences in outcome variables between the intervention group and control group

Outcome Variables	β_0_	β_1_	β_2_	β_3_	β_4_	β_5_	β_6_	β_7_
		T_1 VS._ T_0_	T_2 VS._ T_0_	T_3 VS._ T_0_	IG _VS._ CG	IG × [T_1 VS._ T_0_]	IG × [T_2 VS._ T_0_]	IG × [T_3 VS._ T_0_]
Geriatric Depression	5.23^***^	-0.47	-0.97	-1.32^*^	0.01	-0.45	-1.17	-0.33
State-anxiety	37.59^***^	-3.20^*^	-3.30^*^	-5.14^*^	-0.25	-0.80	-5.80^**^	-3.43
Trait-anxiety	35.87^***^	-2.12	-3.55^**^	-5.90^***^	3.76	-2.01	-6.18^*^	-5.42^**^
Spiritual Well-being	75.38^***^	-1.03	-9.15^***^	-2.85	6.90	5.22	19.20^***^	12.50^**^
Religious well-being	36.48^***^	-1.49	-8.98^***^	-4.23^*^	8.28	3.97	12.50^***^	7.15^*^
Existential well-being	38.89^***^	0.45	-0.18	1.67	-1.37	1.26	6.73^**^	4.80^*^

*M* Mean, *SD* Standard deviation; Regression coefficient β0 is the baseline means for the control group; regression coefficients β1, β2 and β3 indicates the time effect observed for mid-intervention (β1), post-intervention (β2) and 4-week follow-up (β3); regression coefficients β4 is the mean differences in baseline scores between the experimental group and the control group; regression coefficients β5 indicates the mean differences between the experimental group and the control group between β0 and the value at mid-intervention (β5); regression coefficient β6, indicates the mean differences between β0 and the value at post-intervention (β6); last, regression coefficient β7, indicates the mean difference between β0 and the value at 4-week follow-up (β7)

^*^p < 0.05; ^**^p < 0.01; ^***^p < 0.001

## Discussion

Findings of the present study suggest that, compared to usual care, MBEC could potentially improve senior residents' mental health and spiritual well-being. This was clearly demonstrated in the improvement of state- and trait-anxiety and an enhancement of religious and existential well-being. These effects were generally maintained up to four weeks post-intervention, with the exception of state-anxiety. However, no discernible effects on geriatric depression were observed at any post-intervention time points.

Psychological and spiritual distress increase along with aging, especially among those living in long-term residential settings [1]. In the present study, senior residents with disabilities in both the IG and CG had mild depression and slight state- and trait-anxiety. Contrary to expectations, findings of between-group comparisons showed no significant improvement in seniors’ depressive symptoms at all three post-intervention time points. Two possible explanations are suggested for the lack of significant results. First, it is difficult to greatly improve geriatric depression through a short 8-week intervention. In a large cluster-randomized controlled trial, elderly residents’ depressive symptoms did not reduce after even 12 months of exercise intervention [38]. Unlike the time-limited groups of MBSR [23], MBEC requires ongoing group participation [22] so that participants in mindfulness practices are reminded and their participation purposely maintained in order to bring a constant sense of awareness to improve mental health. The other possible explanation is the lack of improvement of depressive symptoms due to an overall low level of depression among participants [21]. In both the IG and CG, almost half of the senior residents’ GDS scores were under 5 points (out of 15 points), indicating mild levels of depression. Their relatively small improvement from the MBEC intervention could be regarded as insignificant. In future studies, seniors with severe depression symptoms may be included to help demonstrate effectiveness.

Anxiety is considered to be a risk factor for the development of depression [39]. Findings of the present study have confirmed that MBEC participants had greater improvements than controls on measures of state- and trait-anxiety upon completion of the intervention, and had long-term effects on reducing trait-anxiety after four weeks. Mindfulness-based intervention has been evidenced to be protective against high levels of stress, anxiety and depression [20]. MBEC shifts the individual’s appraisal of irresolvable bio-psycho-social problems, so life-threatening events are no longer perceived as stressful. Growing evidence has confirmed that mindfulness practice induces changes in both state and trait [40]. However, according to the MBEC curriculum, senior residents in the present study only practiced diaphragmatic breathing, unlike MBSR homework of body scan or mindful eating and movement. Therefore, a lack of sufficient mindfulness practice in the present study is speculated to reduce long-term effects of state-anxiety improvement. Additionally, unlike the similar baseline scores in both groups’ state-anxiety, IG participants had comparatively higher trait-anxiety mean scores at baseline than those in the CG. Such distinction may contribute to the IG’s more significant improvement than that of the CG at 4-week follow-up. Additionally, results of the within-group comparison showed that, compared to baseline, both groups’ depressive symptoms and anxiety levels decreased significantly at two post-intervention time points (T_2_ & T_3_), despite that the improvement in the CG was comparatively less than in the IG. CG participants, in addition to their routine institutional activities, also received a weekly 10- to 15-min visit from the research team to prevent attrition. We suspect that such professional interaction also developed a new social relationship that positively influenced CG participants’ mental health outcomes. In fact, seniors in the CG groups were observed to anticipate these weekly social visits and perceive them as quality sharing time. Because of this effect, a strategy including a short weekly visit from health professionals or volunteers to provide care in terms of social interactions may help to improve senior residents’ mental health, granted that sufficient resources or staff may not be available for MBEC in the residential settings.

Although mindfulness has been verified as an effective strategy for improving the extent of discomfort and spiritual distress in the palliative care setting [41], studies are lacking on the MBEC effects on spiritual well-being for seniors. In the present study, the IG demonstrated a significant increase over the CG in the mean scores of overall and religious and existential subscales for spiritual well-being at both post-intervention and 4-week follow-up. In the MBEC, senior residents’ spirituality is not explicitly targeted [22], but through certain practices such as mindfulness and lovingkindness meditation, a nonjudgmental attitude can be developed toward loss or other challenging situations. Senior residents with disabilities perform mindfulness practices to enhance the awareness of present moment experience, leading to a greater sense of gratitude, compassion and connection with self, others and nature [15]; further the participants in MBEC found meaning through experiential insight and self-transcendence [42]. These attitudinal changes may be key to particularly improving IG participants’ existential well-being.

It is worth mentioning that in the results of within-group comparisons, CG participants’ overall and their religious aspects of spiritual well-being worsened significantly compared to baseline. Spiritual well-being may be seen as a barometer when seniors with disabilities in residential settings face disability and approach death. Spiritual well-being normally declines over time if spiritual care cannot be appropriately and comprehensively provided [32]. Accordingly, findings of the present study highlight the importance of spiritual needs in this population. Feasible and effective spiritual interventions should be integrated into care services to improve residents’ spiritual health in long-term care settings.

Corroborated by our statistical results, participants’ qualitative feedback and PI and RAs’ observations suggest that MBEC was well-received and had positive impacts on mental and spiritual well-being. Participants reported perceived improvement in depressive and anxiety symptoms, with remarks such as “I do feel less anxious in general.” “I realize that being negative is doing nothing to me. I cannot always complain (about the facility). It is what it is now.” They were able to participate more in the long-term care facilities. “After practice (mindfulness), I start to take part in (facility-arranged) activities. Try not to focus on downtime. Have some fun, you know.”

Moreover, participants commented on their changes related to spiritual well-being after mindfulness practice, sharing sentiments, such as “I feel peace and tranquility while practicing mindfulness… I used to feel lonely and abandoned.” “Of course, I missed my own bed at home. I don’t belong here (long-term care facility). But now I know I cannot always look back. I need to accept and start appreciating what I have so far.” Participants’ changes were also reflected in their behavior, as one participant shared, “I used to be so bored here. After mindfulness, I now try to look for things to do (in the facility). Not too bad, some of them.” These findings further emphasize the clinical applicability of MBEC.

### Study limitations

Several limitations of this study are acknowledged. Firstly, we excluded individuals with major clinical depression, and severe sensory or cognitive impairment. This was essential for the practical application of the mindfulness-based interventions, but it potentially narrows the generalizability of our results to different institutional settings serving a broader population. Future research could examine the effectiveness of MBEC for individuals with varying degrees of cognitive and emotional impairments and explore potential adaptations of the mindfulness program. Secondly, although the IG was taken to the consulting room for the MBEC sessions during the institution's regular activity hours, the possibility of contamination between the two groups may not have been completely eliminated. Further studies are suggested using a cluster-randomized controlled study design to select subjects in the two groups. Thirdly, although the research team specifically requested that all participating institutions prevented from providing spiritual care interventions during our trial, existing institutional discrepancies, including routine activities of standard arrangement and resources, may still have created various degrees of biases to MBEC effects, aside from the intervention itself. Future studies may need to document all group activities from each institution while the types, numbers and frequencies of group activities could be treated as covariates in the LMM analysis.

## Conclusion

Results of this study support the feasibility and effectiveness of implementing a mindfulness-based intervention for elder care (MBEC) among seniors with disabilities living in long-term care settings. The 8-week MBEC program, though requiring specialized expertise, has great potential as a cost-effective intervention to selectively benefit certain populations within long-term care facilities by decreasing state- and trait-anxiety and enhancing both religious and existential aspects of spiritual well-being. Future studies are suggested to replicate this investigation with a larger, more diverse sample to increase the generalizability of findings. Additionally, we recommend extending the duration of both the intervention implementation and follow-up periods. Complementary to this, a comprehensive evaluation of the cost-effectiveness of MBEC within these settings should also be considered.

### Relevance for clinical practice

In LTC facilities, seniors with abilities have the potential to adhere to and engage in activities of a nurse-led mindfulness-based intervention. While senior residents’ mental health and spiritual well-being typically decline overtime, results of the present study suggest that MBEC should be integrated into regular LTC institutional routines, particularly for newly relocated residents; health care professional with considerable trainings should be considered to lead on-going mindfulness group sessions in order to maximize MBEC benefits.

## Data Availability

The data that support the findings of this study are available upon request from the corresponding authors.

## Abbreviations

ADLs: Activities of daily living
CG: Control group
LMM: Linear mixed Model
GDS-SF: Geriatric Depression Scale-short form
IRB: Institutional Review Board
IG: Intervention group
LTC: Long-term care
MBEC: Mindfulness-based elder care
MBSR: Mindfulness-based stress reduction
RCT: Randomised controlled trial
RAs: Research assistants
SWBS: Spiritual Well-Being Scale
STAI: State-Trait Anxiety Inventory
SD: Standard deviation
